# Facial Expression Recognition Methods in the Wild Based on Fusion Feature of Attention Mechanism and LBP

**DOI:** 10.3390/s23094204

**Published:** 2023-04-22

**Authors:** Jun Liao, Yuanchang Lin, Tengyun Ma, Songxiying He, Xiaofang Liu, Guotian He

**Affiliations:** 1 Chongqing Institute of Green Intelligent Technology, Chinese Academy of Sciences, Chongqing 400714, China; 2 College of Mechanical Engineering, Chongqing University of Technology, Chongqing 400054, China; 3 Chongqing Key Laboratory of Artificial Intelligence and Service Robot Control Technology, Chongqing Institute of Green Intelligent Technology, Chinese Academy of Sciences, Chongqing 400714, China

**Keywords:** deep learning, facial expression recognition, attention mechanism, LBP features

## Abstract

Facial expression methods play a vital role in human–computer interaction and other fields, but there are factors such as occlusion, illumination, and pose changes in wild facial recognition, as well as category imbalances between different datasets, that result in large variations in recognition rates and low accuracy rates for different categories of facial expression datasets. This study introduces RCL-Net, a method of recognizing wild facial expressions that is based on an attention mechanism and LBP feature fusion. The structure consists of two main branches, namely the ResNet-CBAM residual attention branch and the local binary feature (LBP) extraction branch (RCL-Net). First, by merging the residual network and hybrid attention mechanism, the residual attention network is presented to emphasize the local detail feature information of facial expressions; the significant characteristics of facial expressions are retrieved from both channel and spatial dimensions to build the residual attention classification model. Second, we present a locally improved residual network attention model. LBP features are introduced into the facial expression feature extraction stage in order to extract texture information on expression photographs in order to emphasize facial feature information and enhance the recognition accuracy of the model. Lastly, experimental validation is performed using the FER2013, FERPLUS, CK+, and RAF-DB datasets, and the experimental results demonstrate that the proposed method has superior generalization capability and robustness in the laboratory-controlled environment and field environment compared to the most recent experimental methods.

## 1. Introduction

Facial expressions are one of the most significant indicators of emotional states and intentions in regular human communication [1]. In recent years, facial expression recognition (FER) has played an increasingly vital role in human–computer interaction, intelligent security, multimedia entertainment, autonomous driving, service robots, behavioral psychology, healthcare, and driver tiredness monitoring, among other sectors. Thus, facial expression recognition has become one of the most active areas of study nowadays. Depending on the context in which the datasets are obtained, two methodologies have been developed: FER under controlled settings in the laboratory and FER in the wild. In controlled laboratory contexts, such as CK+ [2], JAFFE [3], MMI [4], and Oulu-CASIA [5], the face pictures are frontal and almost unobstructed, where FER accuracy has achieved amazing results. In the wild facial expressions datasets, such as RAF-DB [6], EmotioNet [7], FERPLUS [8], and AffectNet [9], the recognition accuracy of images collected from the real world in practical applications cannot obtain good results due to occlusions, lighting, pose changes, age, and low-quality facial images. In this context, FER continues to face enormous obstacles.

Traditional methods and deep learning methods are the two categories that can be used to describe approaches to facial expression recognition. Traditional methods rely heavily on manual feature extraction. Histogram of Oriented Gradients (HOG) [10], Local Binary Pattern (LBP) [11], Scale Invariant Feature Transform (SIFT) [12], Non-Negative Matrix Decomposition (NMF) [13], and sparse learning [14] are the most widely used techniques to manually extract facial expression features. Among them, LBP is one of the finest manual feature extraction techniques, with robust picture texture information extraction capabilities and the ability to efficiently adapt to changes in illumination and local rotation. However, these manual feature extraction approaches have a high workload, lengthy stages, low accuracy of facial expression detection in the field, and significant restrictions in terms of practical applications. Niu et al. [15] offered an approach based on a combination of LBP features and an enhanced ORB, which effectively solved the problem of overlapping and redundant feature points in the feature extraction process and produced decent results on multiple experimental controlled datasets, but its output was highly dependent on a priori knowledge and lacked generalization capacity in the field, making it difficult to obtain acceptable results. With the advancement of chip processing power (such as GPU units) and data-driven technologies, deep learning methods in the area of image classification produce superior recognition outcomes than conventional methods. Yang et al. [16] proposed a GAN-based expression intensity enhancement method to solve the problem of low recognition accuracy caused by lighting and pose changes. The method improved the performance of facial expression recognition by combining learning intensity-enhanced facial expressions and expression recognition models to synthesize expression faces embodying high intensity, and the proposed model showed strong applicability. Abiram et al. [17] use an efficient model for embedding a given facial image encoding into the latent vector space of styleGAN for the challenges present in non-frontal poses of facial expression images. By using the latent space vectors of styleGAN to synthesize and retain their facial expressions and non-frontal poses, the synthesized facial images were then passed through a classifier to extract high-level features, and finally, these features were concatenated to perform classification and evaluation. Utilizing an attention mechanism module in a convolutional neural network can increase the ability of convolutional representation, picture classification, etc., which is useful for facial expression detection in the wild, which include images with occlusions and pose changes. Li et al. [18] introduced a convolutional neural network (ACNN) with an attention mechanism for facial expressions that can sense occluded portions of the face and concentrate on the most discriminative uncovered regions. Wang et al. [19] suggested a regional attention network (RAN) that captures the location of essential facial areas for occlusion and position change facial emotion identification.

Despite the significant contribution of deep learning approaches to facial expression identification, the classification accuracy in the field environment still needs to be improved; the key lies in the extraction of facial expression characteristics that are both acceptable and useful. In present facial expression recognition algorithms, the attention mechanism is often performed before the fully connected layer of a CNN network or after certain convolutional layers in a CNN network, and the majority of approaches only consider the spatial or channel dimensions. This research offers an attention mechanism and LBP feature fusion strategy for facial expression recognition, where a hybrid attention mechanism module (CBAM) is added to each residual module to produce a residual attention mechanism module and is embedded in each layer of the network, and where each residual attention mechanism module can improve the feature maps learned in the previous module into m-dimensional feature maps.

The main contributions to this paper are as follows.

(1) Proposed is a residual attention mechanism for facial expression detection in the field that combines a hybrid attention mechanism generated by progressively coupling a channel attention mechanism and a spatial attention mechanism with a residual network. The hybrid attention mechanism may learn the key information of each channel and spatial feature separately to boost the capability of feature expression. Furthermore, inserting the hybrid attention mechanism into the residual network can cause the network to focus on the important regions of facial characteristics.
(2) Proposed is a feature fusion network of a locally increased attention mechanism for fusing the hybrid attention mechanism network model with LBP features. The attention method enables the model to focus more on crucial aspects of facial expressions and suppress irrelevant features, which can substantially increase recognition ability. LBP can capture fine texture details and effectively extract feature information on facial expression images to increase the identification rate of a network.
(3) This paper conducted comprehensive experiments on four public datasets, including both laboratory and wild datasets, to evaluate the proposed approach in this research. The dataset acquired under laboratory conditions is CK+, and the datasets collected in field environments are RAF-DB, FER2013, and FERPLUS. Compared with the current state-of-the-art methods, the suggested method of this paper has superior generalization ability and robustness, and the explanatory power is also improved.

## 2. Related Work

### 2.1. Traditional Methods for FER in the Wild

The traditional facial expression recognition method consists of three steps: face detection, feature extraction, and classification. Face detection technology is rather mature and has been developed into an independent research path and widely utilized in practical applications; thus, the efficacy of facial expression identification method research focuses mostly on the next two steps: feature extraction and classification. There are two types of traditional facial feature extraction algorithms: (1) techniques based on geometric feature extraction, including the active shape model (ASM) and active appearance model (AMM), and (2) techniques based on texture feature extraction, including LBP, HOG, and the Gabor wavelet transform. Due to occlusion and illumination, the detection of facial expressions in the field necessitates more precise feature extraction. Since 2013, FER2013 [20] and EmotiW [21] have organized a series of emotion recognition tournaments to collect huge training datasets of facial expressions from real-world events in order to encourage the application of facial expression recognition technologies [1]. Based on this, the researchers implemented a deep learning model to parallelize the feature extraction and categorization of facial expressions, which significantly increased the recognition accuracy. Tang [22] and Kahou et al. [23] used a deep CNN for feature extraction, which resulted in a significant improvement in expression recognition and won the FER2013 and EmotiW tournaments, respectively. Since then, deep learning has become the predominant technology in the field of expression recognition. Yang et al. [24] suggested a weighted hybrid deep neural network structure to extract facial expression classification features by creating a shallow CNN and a VGG16 grid model pre-trained on ImageNet to conduct facial feature extraction from LBP and face grayscale pictures, the weighted fusion of the two features, and softmax for expression classification output. The experimental findings indicated that the extraction performance of local expression features was enhanced based on the original; however, the increase in accuracy was marginal.

### 2.2. LBP Fusion for Facial Expression Recognition in the Wild

Facial expression identification in the wild must overcome the effects of occlusion and light, which necessitates a greater capacity for image processing and consequently depends more on texture feature extraction. Bazzo et al. [25] proposed a recognition system based on Gabor wavelet features, applying the Gabor kernel to subtract facial expressions of the average neutral face to obtain texture information, which can be used to recognize various facial expressions. In this case, the classifier performance is highly dependent on the quality of texture information extraction. Luo et al. [26] suggested a hybrid approach that combines principal component analysis (PCA) with local binary patterns (LBP). LBP captures local gray features of the mouth region, which contribute the most to facial expression recognition, provides global gray features to aid in facial expression detection, and then applies a support vector machine (SVM) to facial expression recognition. Mehta et al. [27] used a combination of multiple feature extraction algorithms and multiple classification algorithms to determine the optimal combination for emotional intensity recognition. The feature extraction algorithms primarily used LBP, Gabor, and HOG, while the classification algorithms primarily used SVM, random forest (RF), and KNN. Because LBP features have the advantages of grayscale invariance and rotation invariance, they are well-suited for extracting texture information on varying sizes and can solve the issues of occlusion, rotation imbalance, and illumination changes. Experiments indicate that the performance of the fused LBP feature extraction method is significantly superior to the performance of the fused Gabor and HOG methods. Fused LBP feature extraction methods with other methods is currently a significant research approach for facial expression recognition methods.

### 2.3. Attention Mechanism for Facial Expression Recognition Methods

Inspired by the human visual system (HVS), a growing number of computational models of visual attention have been constructed [28] and have proven beneficial to a variety of computer vision tasks. Fernandez et al. [29] suggested an end-to-end attention network model that focuses on the human face and uses a Gaussian spatial representation for emotion identification. The results of experiments indicate that the suggested attention module may effectively increase classification performance. Wang et al. [19] suggested the use of a region attention network (RAN) to handle the posture and occlusion robustness problem in the real world. They used self-attention and relational attention modules in the proposed RAN to aggregate the facial region features of FER in static images and incorporated region bias loss to improve the region weights. Zhu et al. [30] suggested a cascaded attention network that combines an attention mechanism with pyramidal features and is comprised of three modules: a local and multi-scale stereo spatial contextual feature extraction module, a cascaded attention module, and a time series feature extraction module. Its proposed network makes full use of contextual information to compensate for the absence of spatial features, enhances the performance of the attention mechanism, and partially resolves the problem of localization imprecision. Thus, the advantage of the attention mechanism is that it enables the model to prioritize relevant elements while suppressing unimportant ones.

## 3. Proposed Method

### 3.1. Overview

In this section, the network architecture is primarily introduced, and each component is elaborately described. The residual attention mechanism and locally enhanced residual network model are proposed to improve the performance of facial expression image categorization in this research. The residual attention mechanism is composed of the residual neural network, channel attention module, and spatial attention module. Integrating the CBAM attention module with ResNet can enhance classification efficiency.

### 3.2. Residual Attention Mechanism Model

#### 3.2.1. CBAM Attention Module

**Figure 1:**
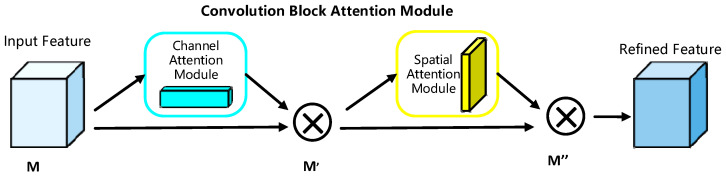
Convolutional block attention module structure diagram.

The attention mechanism is intended to make the neural network pay more attention to the relevant aspects in the input and suppress the non-important features, thus solving the occlusion and non-positive pose problems efficiently. The Convolutional Block Attention Module (CBAM) [31] independently learns the key information of each channel and spatial feature to expand the feature representation and, with the inclusion of the attention mechanism, can improve the model’s efficiency and classification accuracy. The CBAM is made up of two submodules: the Channel Attention Module (CAM) and the Spatial Attention Module (SAM). The CBAM model can be effortlessly integrated into any CNN architecture and trained end-to-end using the basic CNN, which can reduce the number of parameters and amount of processing power required and can be plugged into existing network architectures. Figure 1 depicts the overall design of the CBAM model.

**Figure 2:**
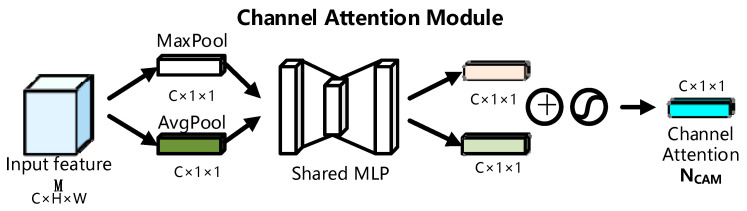
Channel attention module diagram.

Figure 2 depicts the channel attention module’s specifics. After the convolution kernel operation in the CNN, the facial expression picture will generate the image feature matrix (C, H, W), where C signifies the channel feature of the image, H defines the height of the image, and W represents the width of the image. Incorporating the channel attention mechanism into the facial expression classification model can improve the model’s ability to extract global features from facial expression photos. The channel attention module enables the network to prioritize the essential information on facial expression photos while ignoring the rest. The CAM module first compresses the input features $M\in R^{C\times H\times W}$ by global maximum pooling (MaxPool) and global average pooling (AvgPool) in the channel dimension to generate two one-dimensional feature maps, $M_{mp}\in R^{c\times 1\times 1}$ and $M_{ap}\in R^{c\times 1\times 1}$, as shown in Equation (2). Then the two one-dimensional feature maps are sent to a shared multilayer with two hidden layers. The MLP output features are summed element by element ⊕ to merge the output features, and then a sigmoid activation operation is performed to generate the final one-dimension channel attention feature map: $N_{CAM}\in R^{C\times 1\times 1}$.

The computation procedure for the channel attention module can be represented by Equation (2):

(1) $$\begin{array}{l} M_{mp}=MaxPool(M)\\ M_{ap}=AvgPool(M) \end{array}$$

(2) $$\begin{array}{c} N_{CAM}(M)=\sigma (MLP(M_{mp})+MLP(M_{ap}))\\ \;\;\;\;\;\;\;\;\;\;\;\;\;\;\;\;\;\;\;=\sigma (W_{1}(W_{0}(M_{mp}))+W_{1}(W_{0}(M_{ap}))) \end{array}$$

In Equation (2), σ is the sigmoid activation function. In Equation (1), MaxPool is the maximum pooling, and AvgPool is the average pooling. $W_{0}\in R^{C/r\times C}$ is the weight of the first hidden layer in the MLP, and $W_{1}\in R^{C/C\times r}$ is the weight of the second hidden layer in the MLP. For parameter reduction, the number of hidden layers is compressed r times, where r is the reduction ratio and equals 16.

**Figure 3:**
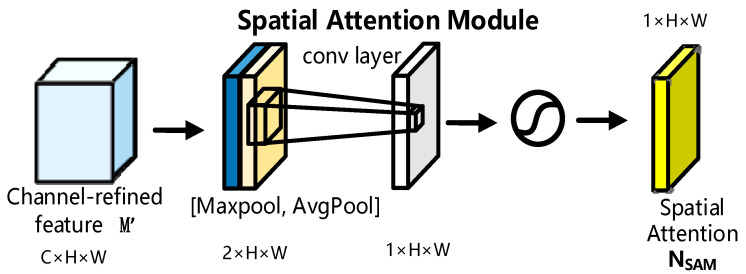
Spatial attention module diagram.

Figure 3 illustrates the spatial attention module’s specifics. The spatial attention module is a crucial supplement to the channel attention module, and its primary function is to extract the core portion of feature information after CAM processing to assign various weights to different parts of the picture in order to concentrate on critical feature information and increase the accuracy of facial expression detection and classification. The SAM module takes the output feature map $M^{\prime }$ of the CAM module as the input feature map. Then in the channel dimension, the input feature map $M^{\prime }$ is processed successively by maximum pooling and average pooling and generates two spatial description matrices of 1 × H × W feature sums, $M_{mp}^{\prime }\in R^{1\times H\times W}$ and $M_{ap}^{\prime }\in R^{1\times H\times W}$, as shown in Equation (4). In this process, not only the contribution from the facial expression image space has been considered, but also the contribution from the global space has been captured. Next, the two spatial description matrices are added together to make a 2 × H × W feature matrix. The number of dimensions is then reduced by 1 × H × W using a 7 × 7 convolution kernel operation. The two-dimensional spatial attention map made by the convolution layer fits the spatial complexity correlation better. Finally, the sigmoid function is used to make a spatial attention feature map, $N_{SAM}\in R^{1\times H\times W}$, that is generated by the sigmoid. Consequently, the addition of a spatial attention module to the classification model can enhance the learning ability of critical areas with high relevance to facial expressions and complement channel attention, thereby enhancing the accuracy of facial expression identification.

The calculation of the spatial attention module can be represented by Equation (4):

(3) $$\begin{array}{l} M_{mp}^{\prime }=MaxPool(M^{\prime })\\ M_{ap}^{\prime }=AvgPool(M^{\prime }) \end{array}$$

(4) $$N_{SAM}(M\prime )=\sigma (Conv([M_{mp}^{\prime };M_{ap}^{\prime }]))$$

In Equation (4), σ is the sigmoid activation function, and Conv represents the convolution operation. The size of the convolution kernel in Equation (4) is 7 × 7. In Equation (3), MaxPool is the maximum pooling, and AvgPool is the average pooling.

#### 3.2.2. Residual Attention Module

**Figure 4:**
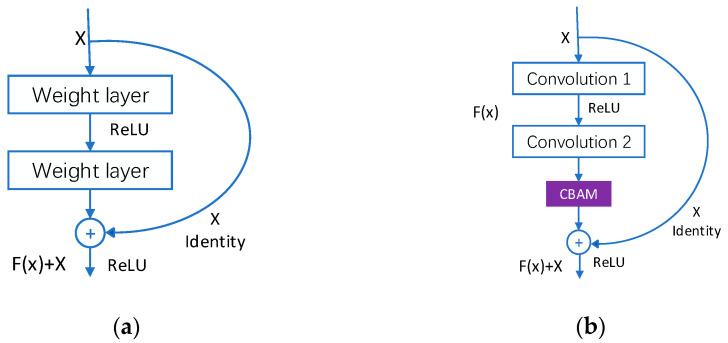
ResNet-CBAM Residual Attention Module. (**a**) Original residual module, (**b**) Residual attention module.

The integration of residual blocks of the attention mechanism necessitates the inclusion of the attention mechanism in the ResNet residual blocks; Figure 4b displays the ResNet-CBAM residual blocks. To eliminate duplicate information and highlight the significant features of the intermediate layers, the CBAM attention mechanism is added to the end structure of each base residual block, and the outcome of the attention is added to the input, thus producing a new feature map. Due to the fact that different layers of the ResNet model use different base residual blocks, the residual base blocks of the attention mechanism are likewise distinct, and this research primarily uses the residual attention module created by the ResNet-18 base residual blocks.

**Figure 5:**
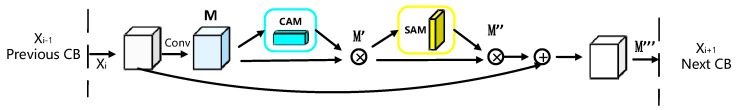
Basic network residual structure integration of CBAM and ResNet. CB—convolutional block; CAM—channel attention module; SAM—spatial attention module.

Figure 5 depicts the model structure of the experiments based on the sequential connection of the CAM and SAM modules to combine the residual module with the CBAM attention mechanism. First, a convolution operation is conducted on the preceding layer’s generated features to produce a feature map $M\in R^{C\times H\times W}$. M acquires the importance of each feature channel via the channel attention module, which increases the model’s weight to focus more on channels associated with facial expressions and suppresses channels with low relevance, thereby obtaining channel attention features $N_{CAM}\in R^{C\times 1\times 1}$. The new feature map $M^{\prime }\in R^{C\times H\times W}$ is produced by multiplying $N_{CAM}(M)$ and the feature map M supplied as input. $M^{\prime }$ is then utilized as the input features of the spatial attention module to obtain the spatial attention feature $N_{SAM}\in R^{1\times H\times W}$. The hybrid feature $M^{\prime \prime }\in R^{C\times H\times W}$ is obtained by multiplying $N_{SAM}$ and the equivalent matrix members of M′. Finally, $M^{\prime \prime }$ is added to the features produced by the previous layer to produce feature $M^{\prime \prime \prime }$, which is the input to the subsequent module. The experimental analysis verifies the integrated position of the CBAM module, hence improving the classification performance of the model.

(5) $$\begin{array}{l} M\prime =N_{CAM}(M)⊗M\\ M\prime \prime =N_{SAM}(M)⊗M\prime \end{array}$$

(6) $$X_{i+1}=X_{i}+M^{\prime \prime }$$

### 3.3. Locally Enhanced Residual Network

#### 3.3.1. Local Binary Patterns

**Figure 6:**
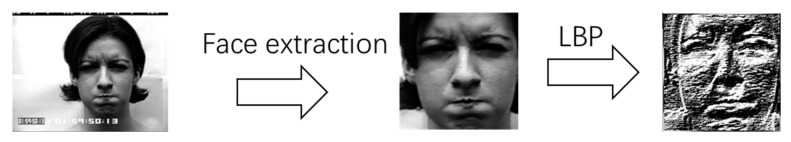
Face extraction and LBP feature extraction.

LBP is one of the most generally used texture pattern descriptors for examining local grain features and is regarded as one of the best methods for texture processing, which is widely employed in image processing. The LBP feature extraction is shown in Figure 6. LBP offers the advantages of grayscale invariance and rotation invariance for illumination-induced changes, making it excellent for extracting texture information of varying sizes and capable of resolving issues such as occlusion, rotation imbalance, and illumination-induced changes. In FER, LBP assists in the recognition of facial expression-related characteristics, such as the eyes, eyebrows, nose, and mouth. Using LBP can successfully extract texture information from a picture and detect minute changes in the face, hence enhancing the performance of a facial expression recognition network. In this study, the LBP operator is specified as a 3 × 3 neighborhood, and the center pixel is utilized as a threshold to compare the grayscale value of the center pixel to those of its eight nearby pixels. The code value is 1 if the bordering pixel value is larger than or equal to the central pixel value; otherwise, it is 0. The resulting 8-bit binary number is encoded, its binary code is translated to a decimal value, and its decimal value is placed as the center pixel value. In the experiments, the improved LBP operator proposed by Ojala et al. [11], namely the circular LBP algorithm, is utilized because the original LBP algorithm can only cover a very small range of 3 × 3 neighborhoods and cannot be applied to the requirement of extracting features of different sizes and frequencies of textures. The circular LBP algorithm expands the 3 × 3 neighborhood to an arbitrary neighborhood and permits the presence of any pair of pixels in a circular neighborhood with a radius. Its definition is given by Equation (7):

(7) $${\mathrm{LBP}}_{N,R}(x_{c},y_{c})=S_{n=1}^{N}s(g_{n}-g_{c})2^{n}$$

In Equation (7), N is the number of sampling points, R is the circle neighborhood sampling radius, $\left(x_{c},y_{c}\right)$ are the center pixel coordinates, $g_{c}$ is the pixel value of the center pixel point, and $g_{n}$ is the pixel value of the neighborhood pixel point. Lastly, S is the symbolic function, and its definition is given by Equation (8):

(8) $$s(x)=\{{}_{0,\;\mathrm{otherwise},}^{1,\;\;if\;x\geq 0,}$$

#### 3.3.2. Network Architecture

**Figure 7:**
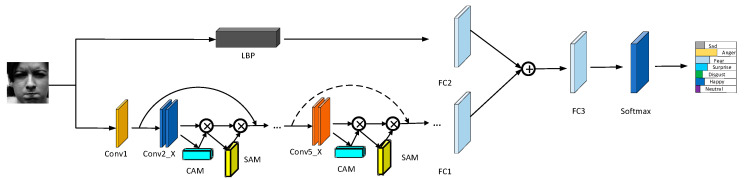
Locally enhanced residual network structure (RCL-Net).

In this research, we present an attention mechanism and LBP feature fusion strategy for the categorization of facial emotions; the resulting RCL-Net model is depicted in Figure 7. The structure is primarily comprised of two branches: the LBP feature extraction branch and the ResNet-CBAM residual attention branch, with the LBP branch extracting local texture data and the ResNet-CBAM branch emphasizing important global characteristics. By passing the input image through the attention branch, the model highlights the key global features and focuses on the important regions of facial expressions, such as the eyes and mouth, and then the extracted features are transformed into feature vectors through the adaptive average pooling layer operation to obtain the feature vector $F_{A}$, and then the input image is passed through the fully connected layer ${FC}_{1}$ and the LBP branch to capture the minute movements of facial expressions. After the local texture features are converted into feature vectors, the feature vector $F_{L}$ is obtained. Then, after the fully connected layer ${FC}_{2}$, the features are fused to obtain $F_{V}=concat({FC}_{1}(F_{A}),{FC}_{2}(F_{L}))$, and the fused features are operated by the fully connected layer ${FC}_{3}$. Finally, the facial expressions are recognized by the softmax classifier, the facial expression classification results are output, and the process is complete. The process formula is shown by Equation (9):

(9) $${F=s(\mathrm{FC}}_{3}{(\mathrm{concat}(\mathrm{FC}}_{1}{(F}_{A}{),\mathrm{FC}}_{2}{(F}_{L}))))$$

In Equation (9), σ is the sigmoid activation function, and F is the facial expression category (7 or 8). The input parameter of ${FC}_{1}$ is 512, and the input parameter of ${FC}_{2}$ and ${FC}_{3}$ are 256 and 14, respectively. The output parameter of 7 or 8 represents the kind of classification result. The ResNet-CBAM residual attention branch considers ResNet18 as the backbone, but the initial 7 × 7 convolution kernel in the original ResNet [32] has been changed into 3 × 3. The maximum pooling layer also has been removed to avoid over-reducing the feature mapping size. The structure of this attention branch consists of a convolutional layer 1, a residual attention module 2, a residual attention module 3, a residual attention module 4, a residual attention module 5, a pooling layer, and a fully connected layer. Where residual attention module 2, residual attention module 3, residual attention module 4, and residual attention module 5 are residual attention modules created by adding an attention mechanism to the convolutional part’s residual blocks. The size of the convolutional kernel is 3 × 3, and the padding is 1 in all convolutional layers. The fused features are then transmitted through a fully-connected layer, which maps the extracted features to the sample space and classifies them using a softmax function to produce classification results.

### 3.4. Loss Function

To reduce overfitting, the label smoothing cross-entropy loss function was used. The label smoothing loss function is shown by Equation (10):

(10) $$\begin{array}{l} loss=(1-\varepsilon )\left[-\sum_{i=1}^{n}P(i)\log_{2}Q(i)\right]+\varepsilon \left[-\sum_{j=1}^{n}P(j)\log_{2}Q(j)/N\right]\\ \;\;\;\;\;\;=(1-\varepsilon )ce(i)+\varepsilon \sum \frac{ce(j)}{N} \end{array}$$

In Equation (10), ε is the smoothing factor, ε∈[0,1]; ce denotes the cross-entropy loss function; and N denotes the number of label categories. The smoothing factor is set as 0.1 in the experiment.

After label smoothing, the label-smoothed distribution is equivalent to adding noise to the actual distribution to prevent the model from being overconfident about the correct label, resulting in a smoother difference between the output values of the predicted positive and negative samples, thereby preventing overfitting and enhancing the model’s generalization ability.

## 4. Experiments

### 4.1. Datasets

**Figure 8:**
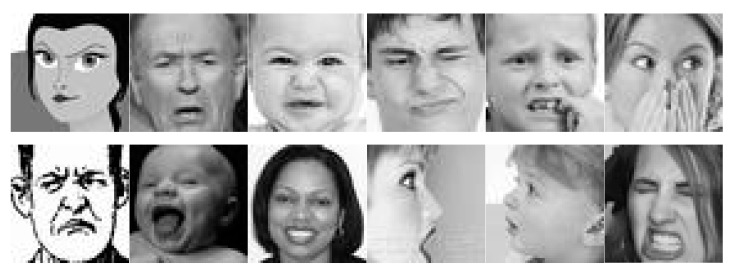
Sample images from FER2013 dataset.

FER2013 Dataset [20]: This dataset consists of 35,887 grayscale face photos with a size of 48 × 48 that are of very low resolution and include a great deal of noise, including motions, partial facial alterations, and occlusions. The collection consists of 28,709 training photos, 3589 private test images, and 3589 public test images depicting seven emotions: anger, contempt, fear, happiness, sadness, surprise, and neutral. The sample images of the FER2013 dataset are shown in Figure 8.

**Figure 9:**
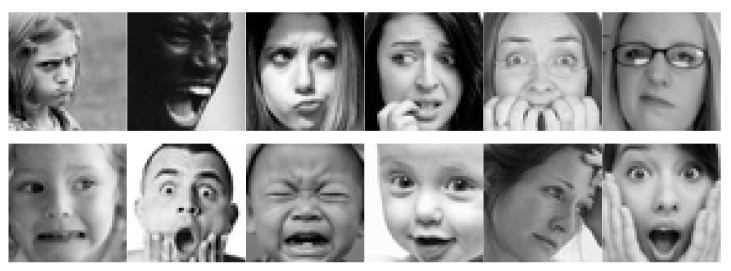
Sample images from FERPLUS dataset.

FERPLUS Dataset [8]: The FERLPUS dataset is expanded from the original FER2013 dataset. Because of the inaccurate annotation of FER2013, Barsoum et al. [8] used crowdsourcing to improve the accuracy of the annotation, and each image from the FERPLUS dataset has 10 labels. In addition to the seven basic expressions in FER2013, contempt, unknown, and non-human face were added to the tags. According to the voting results, we removed the votes that were less than 1 to remove some of the noise. Then we classified the images that received more than half of the total remaining votes on a certain category as that category and removed the unknown and non-face classes. The processed dataset contains 25,060 training pictures, 3153 test images, and 3199 validation images. The sample images of the FERPLUS dataset are shown in Figure 9.

**Figure 10:**
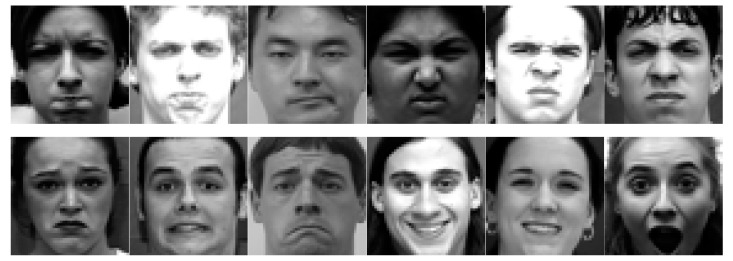
Sample images from CK+ dataset.

CK+ Dataset [2]: The benchmark dataset under a controlled laboratory setting was published in 2010. The dataset contains 593 picture sequences of 123 participants, of which 327 image sequences are classified with facial emotions, including six fundamental expressions (i.e., anger, disgust, fear, happiness, sorrow, and surprise) and disdain. By selecting the last three frames from the identified image sequences, 981 pictures were retrieved as the experimental dataset. The CK+ dataset was separated into two groups, a training set and a test set with a 9:1 ratio. The sample images of the CK+ dataset are shown in Figure 10.

**Figure 11:**
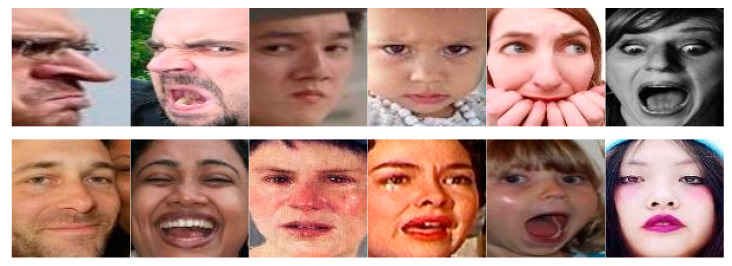
Sample images from RAF-DB dataset.

RAF-DB Dataset [6]: The RAF-DB real-world emotional faces database contains 29,672 RGB images of faces of 100 × 100 size. This dataset contains two different subsets: a single-label subset that contains seven fundamental emotions (surprise, fear, disgust, happiness, sorrow, anger, and neutrality) and a two-label subset that contains twelve types of emotions. Our study employs the single-label subset, which is comprised of 12,271 training images and 3068 test images. The sample images of the RAF-DB dataset are shown in Figure 11.

### 4.2. Implement Details

Data preprocessing: The original image sizes of the FER2013, FERPLUS, CK+, and RAF-DB databases are distinct. To keep the input image size constant, we used the Dlib package to recognize and clip the faces and resize the image pixels to a uniform 100 × 100. We applied data augmentation techniques during the training process to prevent overfitting because the amount of training data was rather small, and no extra data were employed. Cropping random input samples from the center and four corners of the image was completed, followed by horizontal flipping, spanning, rotating, contrast and color perturbation, random masking of the image, and normalizing to [0,1] by dividing each pixel’s gray level by 255.

In addition to data augmentation prior to training, we augmented the training process with the mixup augmentation method [33]. Mixup is a simple but effective strategy for generating training data that reduces overfitting in deep neural networks, and it permits the training of deep neural networks using convex combinations of samples and their labels. In contrast to conventional augmentation approaches, mixup generates virtual training samples by combining training samples. For sentiment classification, we linearly combine photos from the dataset before delivering them to a network. In mixup, two images and their respective labels are randomly selected from the training sample at a time using simple weighted linear interpolation to generate a new sample, which is then fed into the model for training, which is defined by Equation (11).

(11) $$\begin{array}{l} \hat{I}=\lambda I_{i}+(1-\lambda )I_{j}\\ \hat{L}=\lambda L_{i}+(1-\lambda )L_{j} \end{array}$$

In Equation (11), $I_{i}$ and $I_{j}$ denote two random input images, and $L_{i}$ and $L_{j}$ denote their corresponding one-hot encoded labels. For $\lambda \in \left[0,1\right]$, the value of λ is obtained from the distribution of β, and $\lambda \sim \beta \left(\alpha ,\alpha \right)$, among which, $\alpha \in \left(0,\infty \right)$. Consequently, the mixup enlarges the training distribution by synthesizing the data input model using linear interpolation comprising the feature vectors. Using this straightforward data augmentation method, the network’s precision and reliability can be enhanced.

In this paper, we use Pytorch as the frame of deep learning and Python 3.8 as the programming language to conduct experiments in the Windows 10 system environment configured with Intel i7-10700 CPU and NVIDIA GTX3060. In the experiment, SDG is the optimizer, and the initial learning rate is set as 0.9, momentum set as 0.1, weight attenuation set as 0.0001, epochs set as 300, and the batch size of FER2013, FERPLUS, RAF-BD set as 64, and CK+ set as 16. The cross-entropy loss function is utilized for the classification loss function of the network weights.

### 4.3. Evaluation and Comparison

Extensive experiments were conducted on four benchmark expression databases to confirm the validity of this paper’s experimental methodology, including two laboratory-controlled databases, CK+, and three real-world databases, FER2013, FERPLUS, and DAF-DB. The result of this paper has been compared with the most advanced methods.

**Figure 12:**
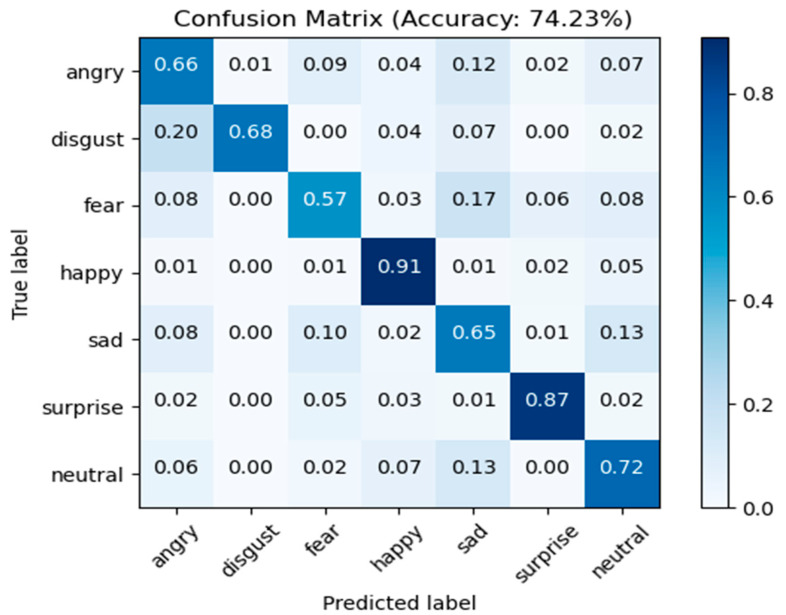
Confusion matrix for FER2013 test dataset.

FER2013 Comparison: The model presented in this paper is compared to several state-of-the-art methods in Table 1 for the FER2013 dataset. The experimental results demonstrate that the method proposed in this paper achieved a recognition rate of 74.23%, which is superior to the most recent methods compared in the table. The method presented in this paper improved recognition rates by 1.83% compared to ResNet [34], by 0.97% compared to the most recent method, Landmark-guided GCNN [35], and by 1.6 percent compared to the ResNet18 baseline network. The confusion matrix of FER2013 on the test set is depicted in Figure 12, where the horizontal coordinates represent the predicted labels, and the vertical coordinates represent the true labels. The accuracy on the diagonal line represents the proportion of correct predictions for each category in Figure 12. The highest recognition rates are 91% and 87% for happy and surprised, while the recognition rates for anger, fear, and sadness are 66%, 57%, and 65%, respectively, due to frequent confusion between these three categories. In addition, the confusion matrix reveals that disgust emotions are frequently misclassified as anger, possibly due to the small number of disgust samples in the original training dataset. In general, the overall recognition rate of the FER2013 dataset is low, primarily because the images of the FER2013 dataset are of low resolution and contain a significant amount of noise.

**Figure 13:**
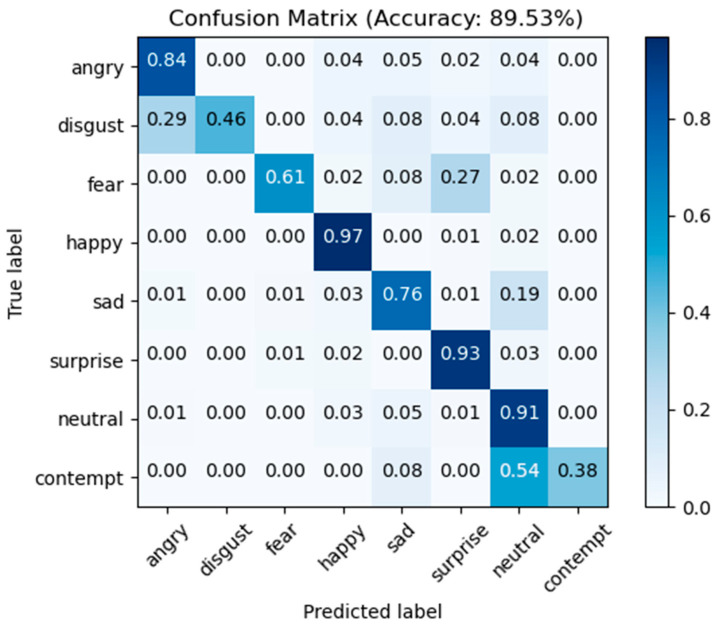
Confusion matrix for FERPLUS test dataset.

FERPLUS Comparison: Table 2 displays the outcomes of a comparison of this paper’s approach using the FERPLUS dataset with other state-of-the-art techniques. We compared our model with other CNN methods, such as ResNet+VGG [42], SENet [43], SHCNN [37], RAN [19], VTFF [44], ADC-Net [45], and the latest methods CERN [46] and A-MoblieNet [47]. The method used in this paper achieved an accuracy of 89.53% using the FERPLUS dataset without the use of pre-trained models, outperforming some of the advanced models in Table 2. Among them, SCN-ResNet18, RAN, and VTFF all utilized ResNet-18 trained using the MS-Celeb-1M face recognition dataset. Compared with the latest methods, CERN and A-MoblieNet, the method used in this paper comparatively improved recognition rates by 1.36% and 1.42%, respectively. Compared with the baseline network ResNet18, the method of this paper improved the recognition rate by 2.69%. The confusion matrix of the model in this paper on the test set is shown in Figure 13. According to the results displayed in Figure 13, the highest recognition accuracy, 97%, is for happy, followed by surprised, neutral, and angry, which are 93%, 91%, and 84%, respectively. The recognition accuracy of contempt is the lowest (38%). We assumed the main reason for this is the insufficient number of samples used for training, making it indistinguishable from neutral. As a result, 54% of contempt is wrongly classified as neutral.

**Figure 14:**
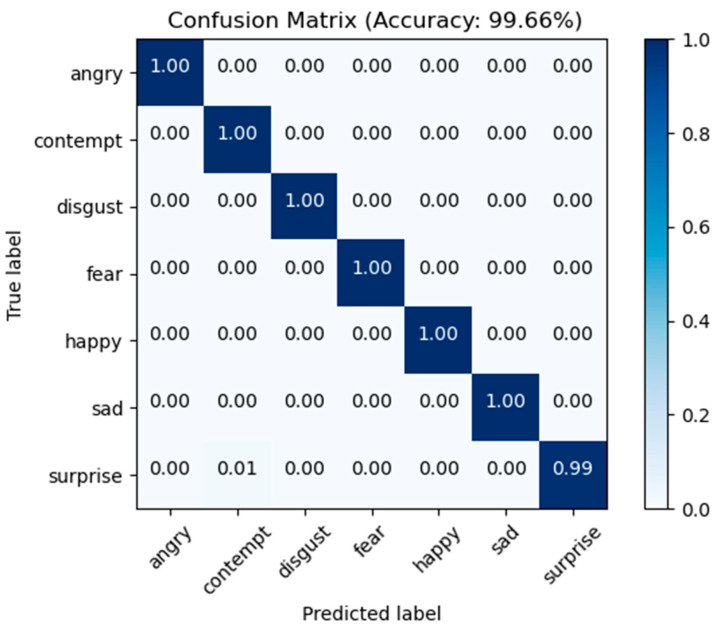
Confusion matrix for CK+ test dataset.

CK+ Comparison: For the CK+ dataset, we compared our method with VGG Net+LSTM [48], SLPM [49], Pre-trained CNN [50], GA-SVM [51], PyFER [52], AC-GAN [53], CNN+SAE [54], and CMCNN [55] methods. The comparison results are shown in Table 3, in which our method achieved 99.66% accuracy using the CK+ dataset. The confusion matrix is depicted in Figure 14 details the categorization results for each of the seven categories of expressions, with the diagonal entries representing the average recognition rate for each category. As demonstrated, the accuracy rates for anger, contempt, disgust, fear, happy, and sad expressions approached one hundred percent, and only a small fraction of astonished expressions were mistakenly identified as disdain expressions. Table 3 demonstrates that the overall recognition accuracy of the CK+ dataset is greater when compared to the other datasets. This is mostly because the CK+ dataset has superior labeling, was gathered under controlled laboratory circumstances, and the image quality is quite high.

**Figure 15:**
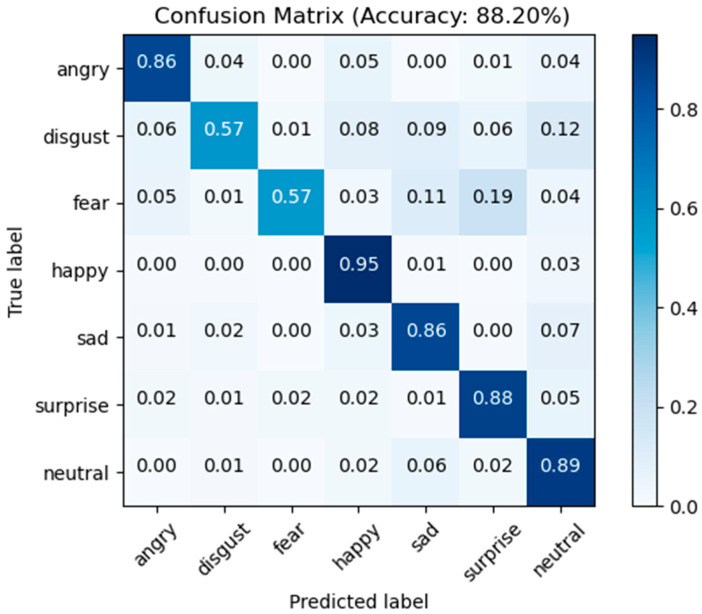
Confusion matrix for RAF-DB test dataset.

RAF-DB Comparison: Table 4 compares the performance of our method with other state-of-the-art methods using the RAF-DB dataset. It can be seen that our proposed method had an accuracy of 88.20% which is higher than most methods but slightly lower than that of MA-Net [56]. The MA-Net method uses ResNet-18 with the MS-Celeb-1M face recognition dataset set of pre-trained models for fine-tuning operations, whereas our method does not undergo model pre-training operations. However, compared with the baseline network ResNet18, our strategy improved the recognition rates by 5.28% because our model places a greater emphasis on the facial expression regions of interest. The following Figure 15 depicts the confusion matrix of our method using the RAF-DB dataset, where the best performance was achieved in recognizing happy expressions with 95% accuracy, followed by neutral, angry, surprised, and sad with 89%, 86%, 84%, and 86%, respectively, as well as disgust and fear with 57% and 57% accuracy, respectively. The sample size of disgust and fear expressions may be too small and insensitive to determine disgust and fear expressions.

### 4.4. Ablation Study

We conducted ablation experiments on the FER2013, FERPLUS, CK+, and RAF-DB datasets to investigate the roles of CBAM and LBP. The first row of Table 5 displays the baseline, i.e., the original ResNet18, with accuracies of 68.29%, 86.84%, 92.29%, and 82.92% for the FER2013, FERPLUS, CK+, and RAF-DB datasets, respectively. After adapting ResNet18 and adding the CBAM module, as shown in the second row, the accuracy for the FER2013 dataset increased by 4.40%. This suggests that adding the attention mechanism improves facial expression identification because the attention mechanism focuses the model’s attention on the most relevant facial expression characteristics. As indicated in the third row, the accuracy increased by 1.54% for the FER2013 dataset, 1.06% for the FERPLUS dataset, 2.52% for the CK+ dataset, and 2.34% for the RAF-DB dataset when LBP feature extraction was introduced to the attention method. This suggests that LBP features that extract texture information on facial expressions can increase network performance. The four datasets improved by 5.94%, 2.69%, 7.37%, and 5.20%, respectively, relative to the baseline.

### 4.5. Visualization

**Figure 16:**
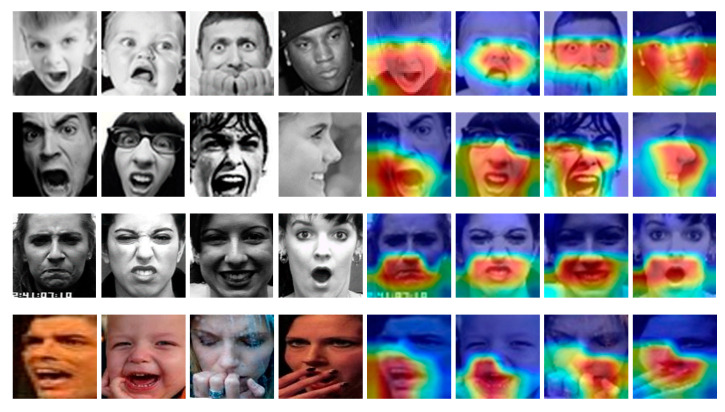
Sample visualization results of different datasets, the left side is the original image graph and the right side is the visualization graph.

In order to evaluate the efficacy of the proposed method, the Grad-CAM visualization technique was utilized to generate expression heat maps, and the resulting heat map samples with and without attention mechanisms were compared across various datasets. The Grad-CAM technique is commonly employed in facial expression recognition to visualize the regions of facial features that the model prioritizes during the classification process. To this end, the original images employed for expression recognition were subjected to the Grad-CAM method, thereby generating heat maps for each expression category. In Figure 16, the heat maps visualized by Grad-CAM for the FER2013, FERPLUS, CK+, and RAF-DB datasets are presented, wherein the original sample image is depicted on the left and the corresponding visualization results on the right. The Grad-CAM method utilizes blue and red effects to generate heat maps around emotional facial expression components, and the analysis indicates that the key features of interest in the expression heat map are the mouth, eyes, nose, cheeks, and other relevant areas.

## 5. Conclusions

In this research, we proposed the RCL-Net model for deep facial expression recognition with an attention mechanism and LBP feature fusion. The model consists primarily of two branches: the LBP feature extraction branch and the ResNet-CBAM residual attention branch. The LBP feature extraction branch captures the minute movements of facial expressions and extracts the texture features of images, while the ResNet-CBAM residual attention branch extracts global features and focuses on the significant regions of facial expressions. Overall, the attention mechanism and LBP feature fusion successfully captured more relevant features, resulting in a substantial improvement in facial expression recognition accuracy. Experimental validation on four publicly accessible facial expression datasets demonstrated that the proposed model achieved an accuracy of 99.66% for the CK+ dataset, 88.20% for the RAF-DB dataset, 74.23% for the FER2013 dataset, and 89.50% for the FERPLUS dataset.

The experimental results demonstrate that, compared to the most recent methods, the method presented in this paper resolves the issues of imbalance between cross-library datasets and low recognition rate; it has superior generalization ability and robustness in the laboratory and natural environments; and it demonstrates that the method of an augmented residual attention model incorporating LBP features and an attention mechanism is feasible.

## Figures and Tables

**Table 1:** Comparison of accuracy of different models with FER2013 dataset.

Method	Year	Accuracy (%)
ResNet [34]	2016	72.40
CPC [36]	2018	71.35
SHCNN [37]	2019	69.10
Fa-Net [38]	2019	71.10
BReG-NeXt-50 [39]	2020	71.53
DisEmoNet [40]	2021	71.72
VGGNet [41]	2021	73.28
Landmark-guided GCNN [35]	2022	73.26
Ours	2022	74.23

**Table 2:** Comparison of accuracy of different models with FERPLUS dataset.

Method	Year	Accuracy (%)
ResNet+VGG [42]	2017	87.40
SENet [43]	2018	88.80
SHCNN [37]	2019	86.54
RAN [19]	2020	88.55
VTFF [44]	2021	88.81
ADC-Net [45]	2021	88.90
CERN [46]	2022	88.17
A-MobileNet [47]	2022	88.11
Ours	2022	89.53

**Table 3:** Comparison of accuracy of different models with CK+ dataset.

Method	Year	Accuracy (%)
VGG Net+LSTM [48]	2017	97.2
SLPM [49]	2018	96.1
Pre-trained CNN [50]	2019	95.29
GA-SVM [51]	2020	97.59
PyFER [52]	2020	96.3
AC-GAN [53]	2021	97.39
CNN+SAE [54]	2022	98.65
CMCNN [55]	2022	98.33
Ours	2022	99.66

**Table 4:** Comparison of accuracy of different models with RAF-DB dataset.

Method	Year	Accuracy (%)
gACNN [18]	2018	85.07
APM-VGG [57]	2019	85.17
MA-Net [56]	2020	88.42
DisEmoNet [40]	2020	83.78
RAN [19]	2020	86.90
VIFF [44]	2021	88.14
A-MobileNet [47]	2022	84.49
CMCNN [55]	2022	85.22
Ours	2022	88.20

**Table 5:** Ablation experiments on different datasets.

Model	FER2013	FERPLUS	CK+	RAF-DB
Baseline	68.29%	86.84%	92.29%	82.92%
ResNet+CBAM	72.69%	88.47%	97.14%	85.82%
ResNet+CBAM+LBP	74.23%	89.53%	99.66%	88.20%

## Data Availability

Not applicable.
